# The University Münster model surgery system for orthognathic surgery - The digital update

**DOI:** 10.1186/s13005-021-00278-y

**Published:** 2021-07-23

**Authors:** Thomas Stamm, Dennis Böttcher, Johannes Kleinheinz

**Affiliations:** 1 Senior Lecturer, Department Orthodontics, University of Münster, Albert-Schweitzer-Campus 1, Gebäude W 30, Münster, D-48149 Germany; 2 Orthodontist, Department Orthodontics, University of Münster, Albert-Schweitzer-Campus 1, Gebäude W 30, Münster, D-48149 Germany; 3 Head of Department, Department of Cranio-Maxillofacial Surgery, University Hospital Münster, Albert-Schweitzer-Campus 1, Gebäude W 30, Münster, D-48149 Germany

**Keywords:** Orthognathic surgery, Virtual planning, Orthodontics

## Abstract

**Aim:**

The aim of this work is to present a digital methodology of a conventional articulator based planning protocol.

**Methods:**

The digital counterpart consists of intra-oral scans (3Shape) and a free available 3D mesh software (Meshmixer, Autodesk). The maxillary position in relation to the reference plane used and the arbitrary hinge axis were determined mathematically from landmarks on cephalometric x-rays and frontal photographs. Distances and angles were calculated to virtually mount the digital jaws in Meshmixer’s wold frame. Virtual planning is done by cloning and moving the jaws according to the preliminary surgery plan. The spatial movements of the jaws are measured by attached reference points.

**Results:**

This digital approach eliminate the need for articulator hardware and laboratory plaster work. It enables all planning scenarios as they are also possible with conventional plaster-based procedures. The method is time-saving, practical and cost-effective. Standard dimensions of articulators and face-bows have been incorporated in the implementation. This reduction of individual patient values puts the accuracy of the presented method within the range of conventional model surgery.

**Conclusion:**

Arbitrary planning will continue to have its place in orthognathic surgery, especially when digital methods can improve the overall process. The method presented can be seen as a cost-effective alternative for patients who do not require technically complex planning.

## Background

The essential factors of a successfully treated orthognathic surgery case are a) “ortho”-gnathic jaw position, b) facial improvement, c) good occlusion, and d) balanced function. Interdisciplinary collaboration between surgeons and orthodontists strives to meet these factors. Without a doubt, each physician involved aims to achieve an optimum in his field. This also applies to surgery planning, where the orthodontist wants to achieve a treatable postoperative situation and the surgeon wants to perform a cause-oriented osteotomy to move and stabilize the jaws or segments in the planned final position.

Although articulators were never designed for orthognathic surgery [1] they have been used for decades. Articulators have fixed, mean-value dimensions with respect to the intercondylar distance. The face-bow places the occlusion in relation to the axis of the articulator, which does not correspond to the individual hinge axis of the patient. This is called arbitrary mounting. In the following, planning based on fixed average articulator dimensions is referred to as ‘arbitrary planning’.

Surgical planning has developed fundamentally different from analogue planning and new waiverless/splintless surgery with patient specific implants seem to be emerging as the future gold standard. However, high costs and technical demands of the new technology make these inaccessible to many patients [2]. Moreover, the significant improvement of the results and benefit for the patient in comparison with the analog technique is still not proven. It is therefore necessary to offer a cost-effective method without having to forego the benefits of digital workflows.

Intraoral scanners are of particular importance in surgery planning. Due to their high accuracy and practicability, they could replace impressions [3], as well as scattered dental morphology in cone-beam computed tomographs [4]. They are therefore applicable to create composite virtual skull models and conventional planning procedures. Freely available, high-precision software from the engineering industry pave the way for experimenting with cost-effective planning alternatives.

Another aspect for the transfer of conventional planning into a digital workflow is the enormous experience gained in decades of surgery, which represents a wealth of knowledge that should not be discarded. Known errors and inaccuracies are more likely to be accounted for and contribute to faster and more practical implementation. The knowledge and the indication-related application of all possible surgical techniques is mandatory for each planning system. This fundamental clinical experience cannot be replaced by digital algorithms. The aim of this work is therefore to describe a method of incorporating the coordinate system and rotation axis of a conventional semi-adjustable articulator into the digital three-dimensional surgical planning.

## Method

The authors’ methodology is based on the principles of model surgery planning on dental casts described by Ehmer et al. [5–9]. This system is part of the standard planning of orthognathic surgery at the University of Münster that includes the fabrication of intermediate and final gnathological splints using the semi-adjustable articulator SAM 2P (SAM Präzisionstechnik GmbH, Gauting, Germany).

In the digital version, the impressions are replaced by intra-oral scans (3Shape A/S, Holmens Kanal 7, 1060 Kopenhagen, Dänemark) and the patient’s individual maxillary position in relation to the used reference plane as well as the arbitrary hinge axis are virtually combined with the 3D mesh software Meshmixer (Autodesk, Inc. 111 McInnis Parkway San Rafael, CA 94903 USA). With this digital counterpart, face-bow, face-bow bite fork and articulator can be omitted. Further diagnostic records for digital planning are the clinical examination, a panoramic x-ray, a cephalometric x-ray and special profile and frontal photographs. It is important to note that the goal of this method is a digital representation of the planning using the SAM 2P including the special hardware necessary for orthognathic surgery [9]. Therefore, this system is working arbitrary with the conditions of an anatomic face-bow and standard articulator dimensions.

**Fig. 1:**
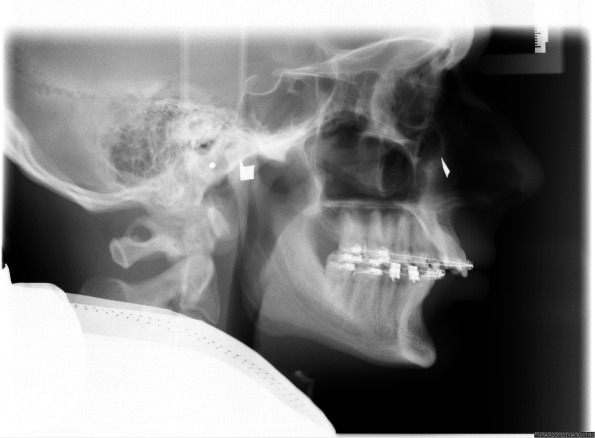
Clinical approach to the determination of the axis orbital plane. The individual hinge axis and the right palpable infraorbital rim were indicated with metal markers on the patient’s skin before a cephalometric radiograph was obtained. Square and triangular markers were used to distinguish between left and right side

The SAM’s (articulator and face-bow) reference plane is the axis orbital plane (AOP) which is also the common reference plane for two dimensional cephalometric and conventional articulator-based model surgery in a couple of schemes [10–13]. Although there are some techniques for localization of the AOP in cephalometric radiographs [14–16] we used a clinical approach. This approach is based on planned cases, where it was necessary to project the individual hinge axis and the palpable infraorbital rim on the cephalometric x-ray to perform a two-dimensional planning according to individual measurements. For this purpose, the positions were marked with metal markers on the patient’s skin before a x-ray was taken (Fig. 1).

A retrospective analysis of several marked x-rays from previous cases revealed that the geometric relations between the maxillary occlusal plane and the face-bow plane can be calculated by using four cephalometric landmarks: Pi (Porion inferior, deepest point on the lower border of the meatus acusticus externus), Ns (nasal support of the face-bow), Ie (upper incisor edge) and Dc (distobuccal cusp of the first permanent upper molar). For double contours (right/left), the average is taken. Ie and Dc form the maxillary occlusal plane. For simplification in daily use, a script was written that uses the free software ImageJ [17] to calculate the necessary angles and distances for transmission to Meshmixer. The theoretical background is as follows.

### Calculation of the maxillary position in the sagittal plane

**Fig. 2:**
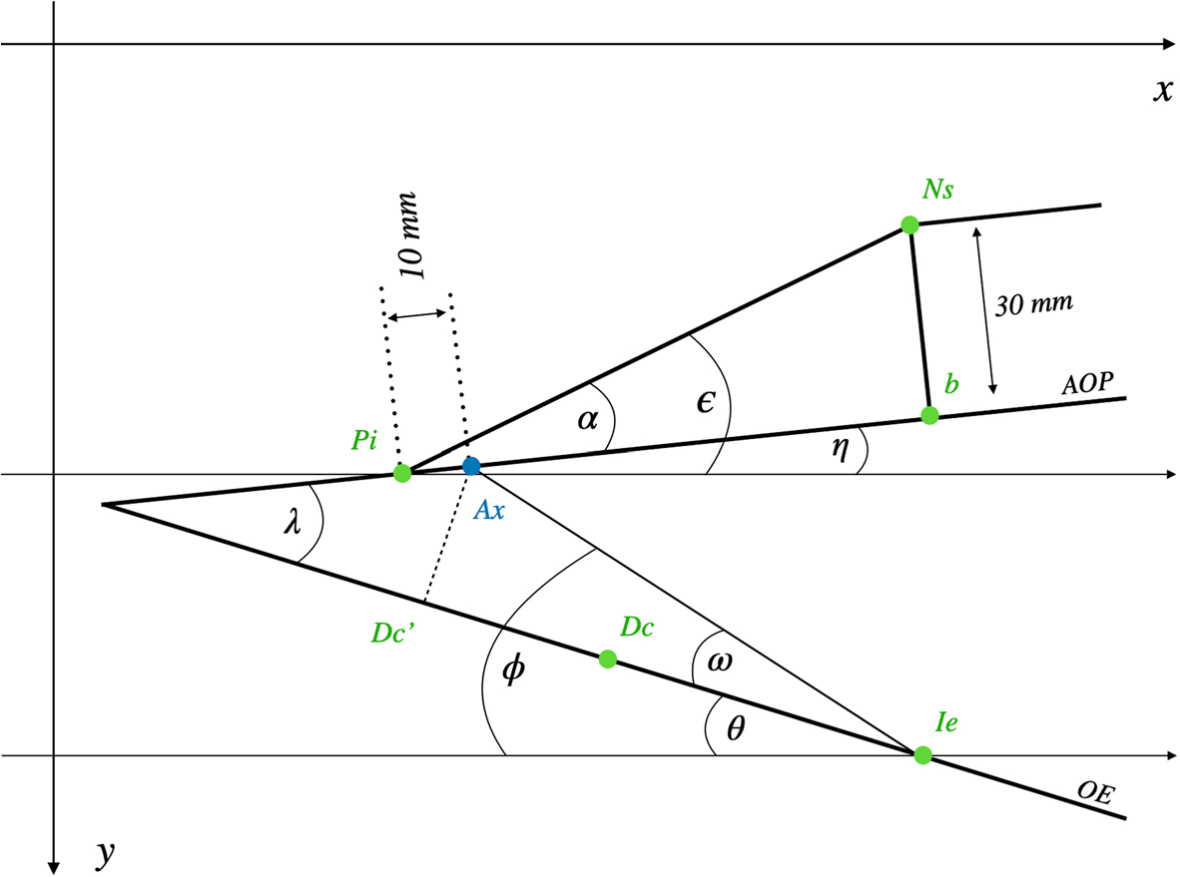
Schematic representation for calculating the angles and distances between the face-bow, articulator axis and occlusal plane. Pi = Porion inferior; Ax = hinge axis position of the SAM (10 mm anterior of the ear rods); Ns = Nasal rest of the face-bow; AOP = axis orbital plane represented by the face-bow; OE = occlusal plane; Ie = upper incisor edge; Dc = distobuccal cusp of the first permanent upper molar; Dc’ = section of a perpendicular line from Ax to the occlusal plane

A schematic representation for calculating the angles and distances between the face-bow, articulator axis and occlusal plane is presented in Fig. 2. All calculations are performed with script language of ImageJ where standard tools like *Set scale*, *Brightness/Contrast* and *Window/Level* were used.

After importing and scaling of the cephalometric radiograph the above mentioned landmarks are placed with ImageJ’s *multi-point* tool. The first calculation concerns the right-angled triangle between Pi, Ns and b, where b is the point on the face-bow plane perpendicular to Ns. The distance Ns-b is constant (30*mm*) due to the face-bow hardware. The distances Pi-Ns, Pi-b and the angle *α* between Pi-Ns and Pi-b are calculated as follows. The calculations shown here refer to the coordinate system of ImageJ, where the origin is in the upper left corner.

1 $$ \lVert Pi-Ns\rVert =\sqrt{\left(x_{Ns}-x_{Pi}\right)^{2}+\left(y_{Ns}-y_{Pi}\right)^{2}}  $$

2 $$ \alpha = \arcsin \left(\frac{ \lVert Ns-b\rVert }{ \lVert Pi-Ns\rVert }\right)  $$

3 $$ \lVert Pi-b\rVert = cos \alpha \cdot \lVert Pi-Ns\rVert  $$

The angle *ε* between *Pi*−*Ns* and a horizontal line could be calculated by the slope of the Pi-Ns:

4 $$ m_{Pi-Ns} = \frac{y_{Ns}-y_{Pi}}{x_{Ns}-x_{Pi}}  $$

5 $$ \epsilon = \arctan (m_{Pi-Ns})  $$

The angle *η* between *Pi*−*b* and a horizontal line is therefore:

6 $$ \eta = \epsilon - \alpha  $$

The locate the SAM’s hinge axis on the angled face-bow plane its slope must be calculated:

7 $$ m_{Pi-b} = \tan \eta  $$

The angle *θ* between the occlusal plane and a horizontal line can be calculated by the slope of *Ie*−*Dc*:

8 $$ \theta = \arctan \frac{y_{Ie}-y_{Dc}}{x_{Ie}-x_{Dc}}  $$

Thus, the angle *λ* between face-bow and occlusal plane is:

9 $$ \lambda = \theta + \eta  $$

### Calculation of the arbitrary hinge axis position

The ear rods of the SAM face-bow are located in a distance of 10 mm behind the articulator’s hinge axis during mounting of plaster casts. To transfer this position into Meshmixer the following calculations are necessary: i) the location of the articulator’s axis (Ax) on the face-bow plane, ii) the distance from Ax perpendicular to the section (*Dc*^′^) on the occlusal plane and iii) the distance from *Dc*^′^ to the upper incisor edge (Ie).

The coordinates of *Ax* 10 mm anterior of *Pi* on the face-bow plane are: *x*_*Ax*_= cos*α*·10 and *y*_*Ax*_= sin*α*·10. With the coordinates of point *Ie* (*x*_*Ie*_,*y*_*Ie*_) the angle *ϕ* between *Ax*−*Ie* and a horizontal line could be calculated by:

10 $$ \phi = \arctan \frac{y_{Ie}-y_{Ax}}{x_{Ie}-x_{Ax}}  $$

The angle *ω* between *Ax*−*Ie* and the occlusal plane is *ϕ*−*θ*. The distance between the articulator’s axis and the upper incisor edge could be calculated by

11 $$ \lVert Ax-Ie\rVert = \frac{y_{Ie}-y_{Ax}}{\sin \phi}  $$

With the disctance *Ax*−*Ie* and the angle *ω* the distance from *Ax* perpendicular to the occlusal plane could be calculated by

12 $$ Ax_{ppd} = \sin \omega \cdot Ax - Ie  $$

Therefore, the distance on the occlusal plane from *Ie* to *Ax*_*ppd*_ is

13 $$ \rVert Ie - Ax_{ppd}\lVert = \frac{Ax_{ppd}}{\tan \omega}  $$

**Fig. 3:**
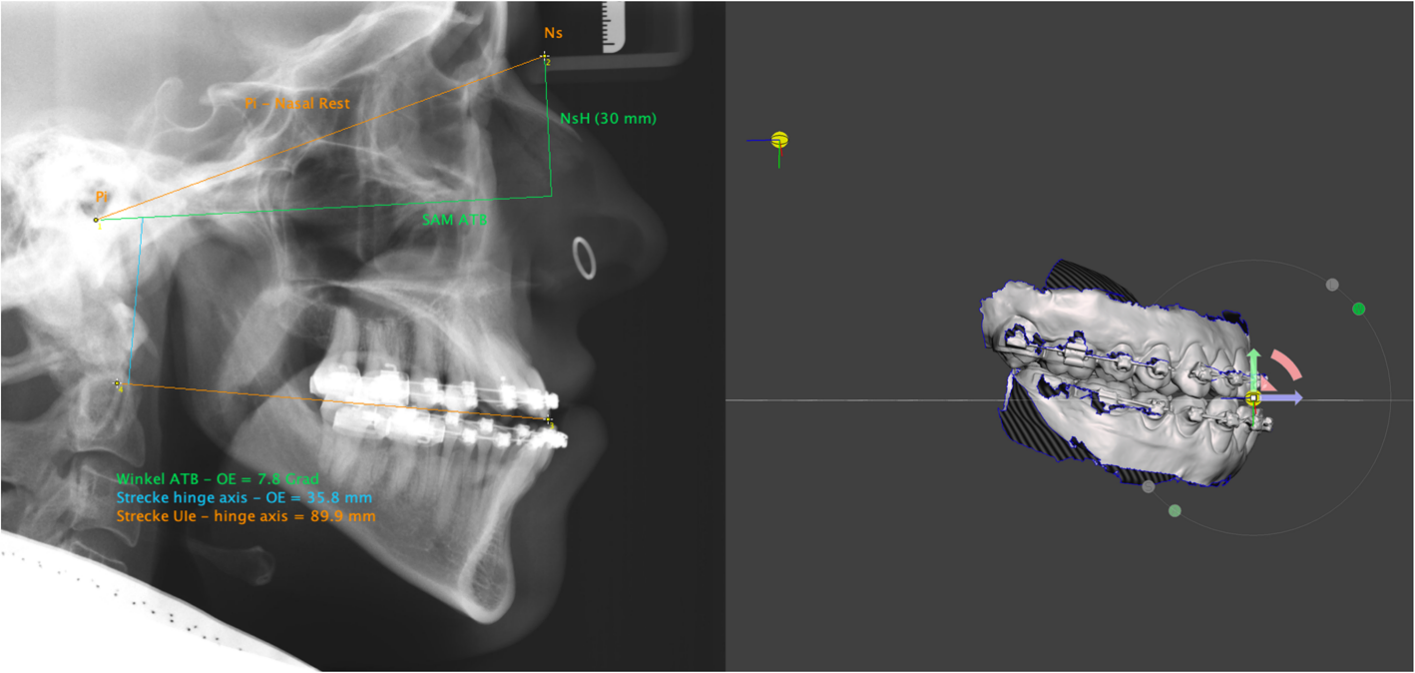
Left: The following calculations are projected on the cephalometric x-ray: Angle between upper occlusal plane and AOP (7.8^∘^), distance from the articulator’s hinge axis perpendicular to the upper occlusal plane (35.8*mm*), and the distance from the latter intersection to the upper incisor edge (89.9*mm*). Right: Movement of the hinge axis pivot according to the calculated distances following clock wise rotation of the all objects around upper incisor edge. Upper and lower jaw are thereby arbitrarily aligned to the AOP in Meshmixer’s world frame

The distances from Eqs. 11 and 12 are the important distances to place the hinge axis in relation to the maxillary scan within the Meshmixer software. A graphical representation of the calculations is projected on the cephalometric x-ray (Fig. 3, left).

### Transverse inclination of the occlusal plane

**Fig. 4:**
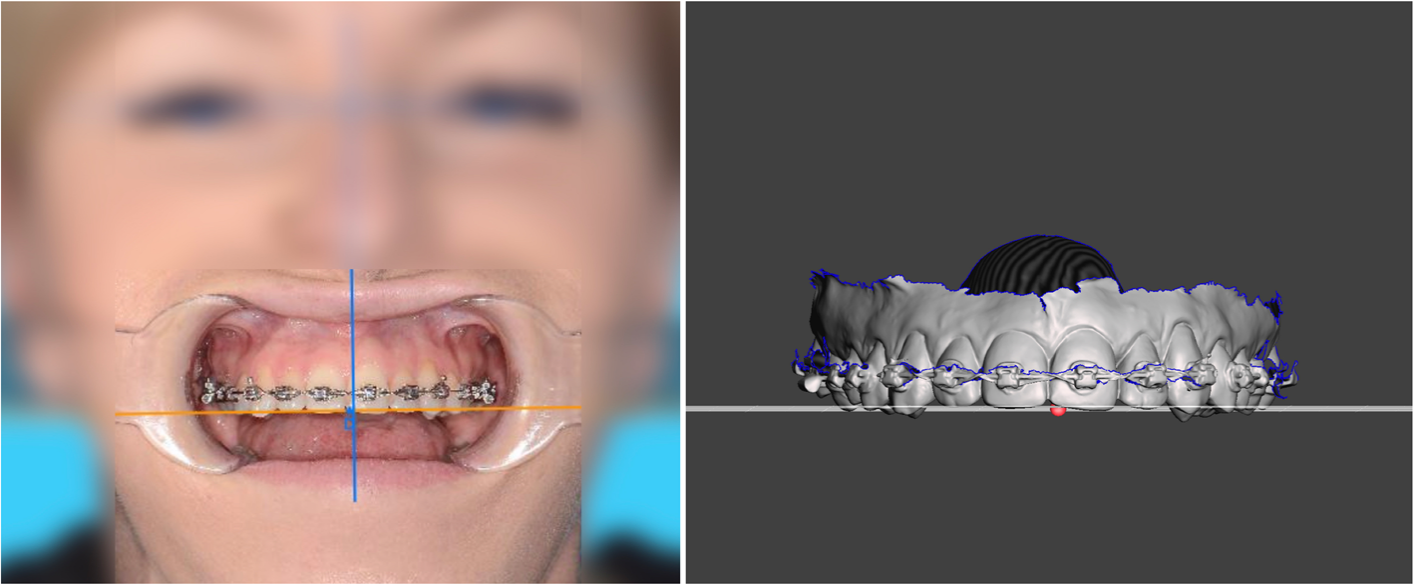
Left: Frontal image of the patient, gazing forward with mouth open and cheek retractors in place. The head must be tilted forward or backward so that the aligned teeth are in one plane. A line parallel to the interpupillary line is drawn to assess the maxillary occlusal plane. Right: The upper jaw is aligned to Meshmixer’s ground-plane grid according to the reference lines of the frontal image. The dental midline is slightly shifted to the right of the mid-sagittal plane (red dot)

The assessment of the occlusal plane in transverse direction is done by means of the interpupillary line. In the absence of trauma, tumors, or syndromal conditions, it can be assumed that in the majority of skeletal malocclusions (class II, class III, open bite), the orbits are not asymmetrically positioned and that both pupils could be used to assess the facial midline and inclination of the occlusal plane. Since no scaling is required for angle measurements, a frontal image of the patient, gazing forward with mouth open and cheek retractors in place is sufficient to assess the occlusal plane (Fig. 4, left).

### Data transfer to the mesh software

**Fig. 5:**
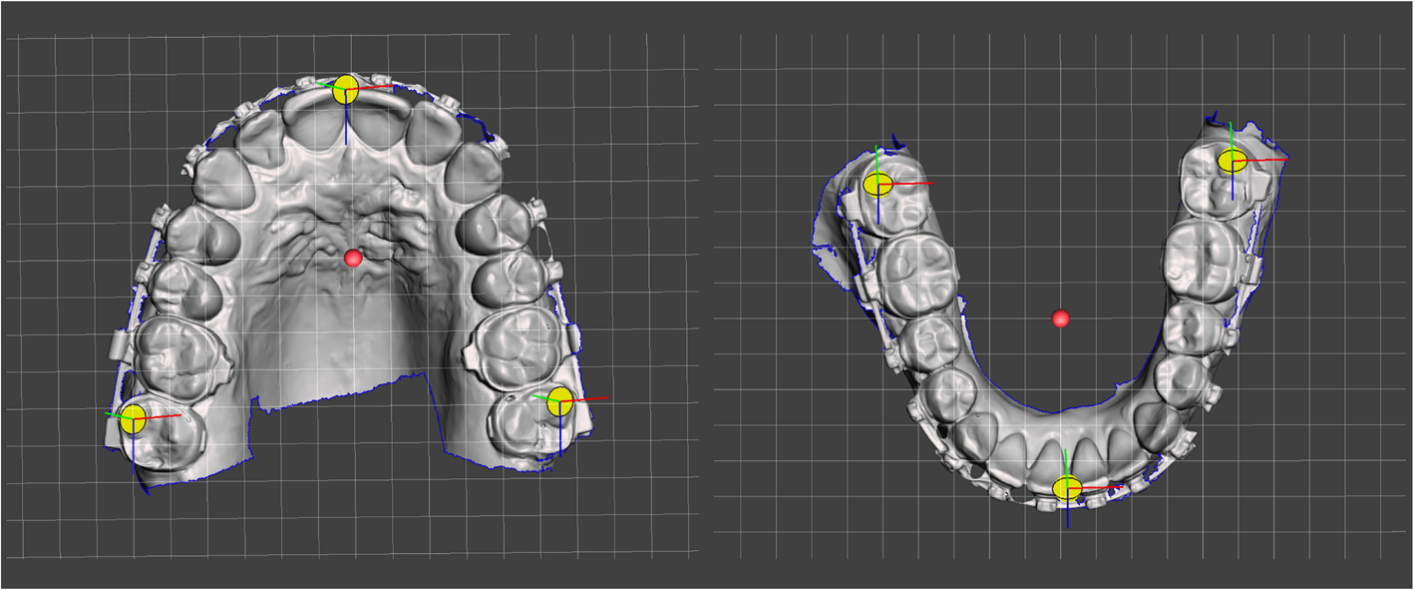
Pivots are used as measurement points on the surface of the jaws, which bind a 3D location to an object

The intraoral scans are imported into Meshmixer and the maxilla is aligned to the world frame’s ground-plane grid. The world frame is the canonical axis-aligned coordinate system, where Y always points upwards, and X always points to the right. In the horizontal plane the maxilla is symmetrically oriented along the palatal suture to the midsagittal plane and with its occlusal plane parallel to the grid. Dental midline deviation must be taken into account (Fig. 4, right), as well as mesial- or distal-positions of the posterior teeth in case of asymmetric arches (Fig. 5). Possible tilts of the occlusal plane around the z-axis (antero-posterior) can be adjusted to the exact degree (Fig. 4, right). The mandible is registered to the maxilla through the bite-scan and is moved passively when the maxilla is positioned.

Once the scans are ideally aligned to the planes measuring points should be set. Meshmixer provides pivots for this purpose. Pivots are persistent objects in a scene that allow to store or to bound a 3D location to an object. The number and localisation of the desired measuring points can be freely selected. Essential for the concept presented here, however, is a pivot on the upper incisor edge, ideally in the plane of the skeletal midline. It is not necessary to choose reproducible surfaces, because the pivots are placed only once and cloned each time for further processing.

The next step is the alignment in the sagittal plane according to the calculations obtained from the cephalometric radiograph. For this the pivot on the upper incisor edge is cloned and moved distally along the ground plane grid (which coincides with the occlusal plane in this phase) by the amount of the calculation from Eq. 13. Then it is moved up by the amount of the calculation from Eq. 12. Now the pivot represents the articulator’s hinge axis in relation to the maxilla. Any object bounded to this pivot could be rotated around the pivot’s axis. To complete the virtual mounting jaws and pivots must be angulated to the AOP. To do this, all objects in the scene are bound together and rotated around the x-axis of the upper incisor pivot (Fig. 3, right). The size of the rotation corresponds to the result from Eq. 9. With this rotation, the ground-plane grid becomes a parallel to the AOP and the scans are in correct relation to this reference plane and to the arbitrary hinge axis. The scene is ready for virtual planning.

### Virtual planning

After generating the preliminary surgical plan, the STL jaws including bounded pivots are cloned and used for the desired movements. The original STLs remain in their initial *unoperated* positions and are used for calculation of the distances between *pre-* and *postoperative* position. In the following, *’jaw’* always means the corresponding STL file with the measurement pivots bound to it.

**Fig. 6:**
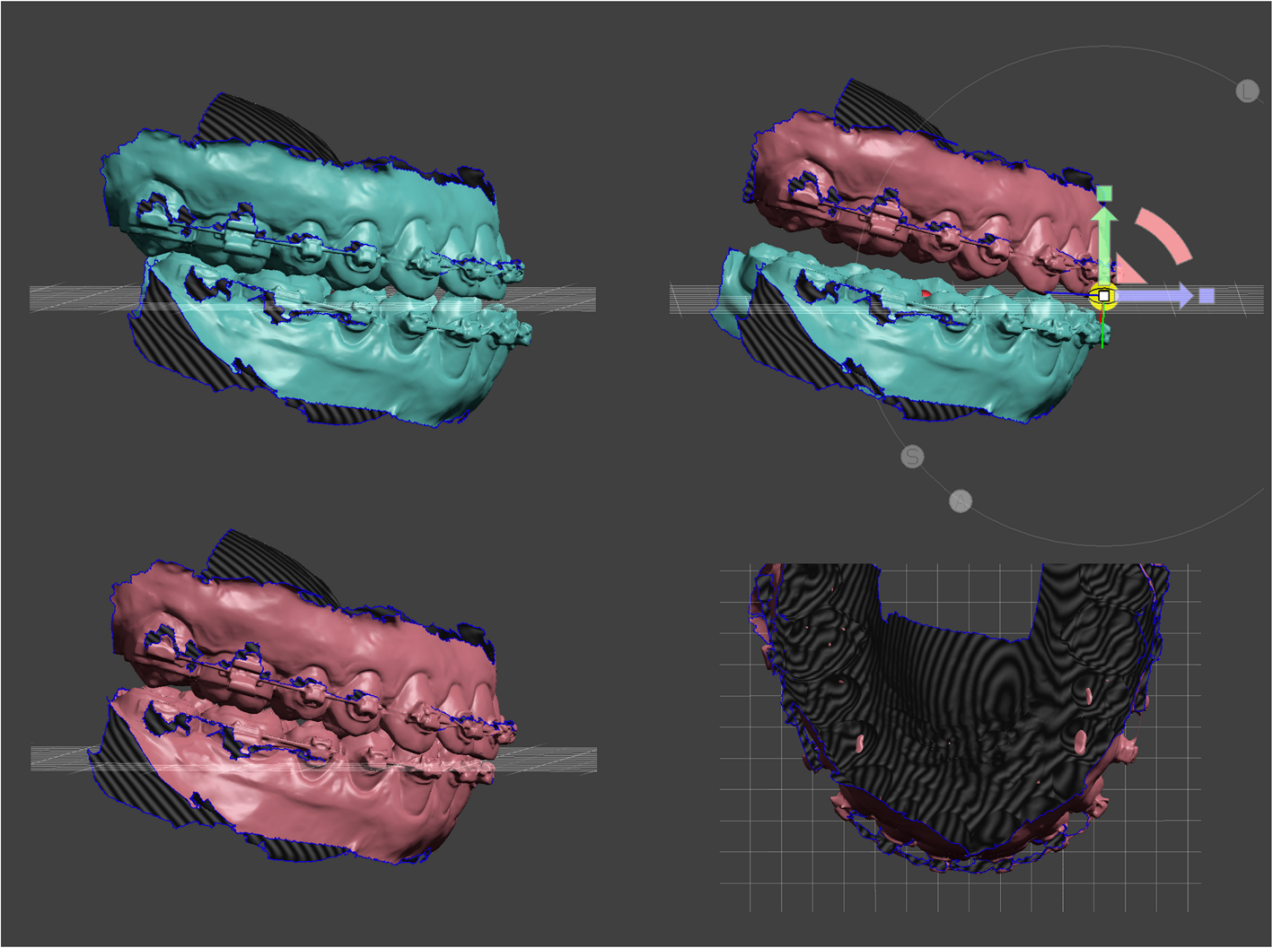
The jaws are color-coded for surgery simulation to visually indicate the pre- and postoperative position. Upper left: Initial situation prior to bimaxillary surgery. Upper right: Cloned upper jaw advanced with posterior impaction. The center of transformation is located in the center of the pivot (widget) at the upper incisor edge. Lower left: Set back of the cloned lower jaw in final occlusion. Lower right: Top view of merging areas between upper and lower occlusal meshes. Early contacts occur in these areas, which must be taken into account during postoperative orthodontic treatment

In general, bi-maxillary surgical planning starts by cloning and moving the upper jaw in its new position. This can be done in a simple way with Meshmixwer’s 3D transform widget. The widget is located in the center of transformation of the jaw. Selecting one of the pivots switches the center of transformation to the center of the pivot which could be helpful for desired rotational movements (Fig. 6, upper right). More precise movements can be made with the transform tool property panel in which the numeric-value fields can be directly edited. This is advantageous for fine-tuning the final position.

**Fig. 7:**
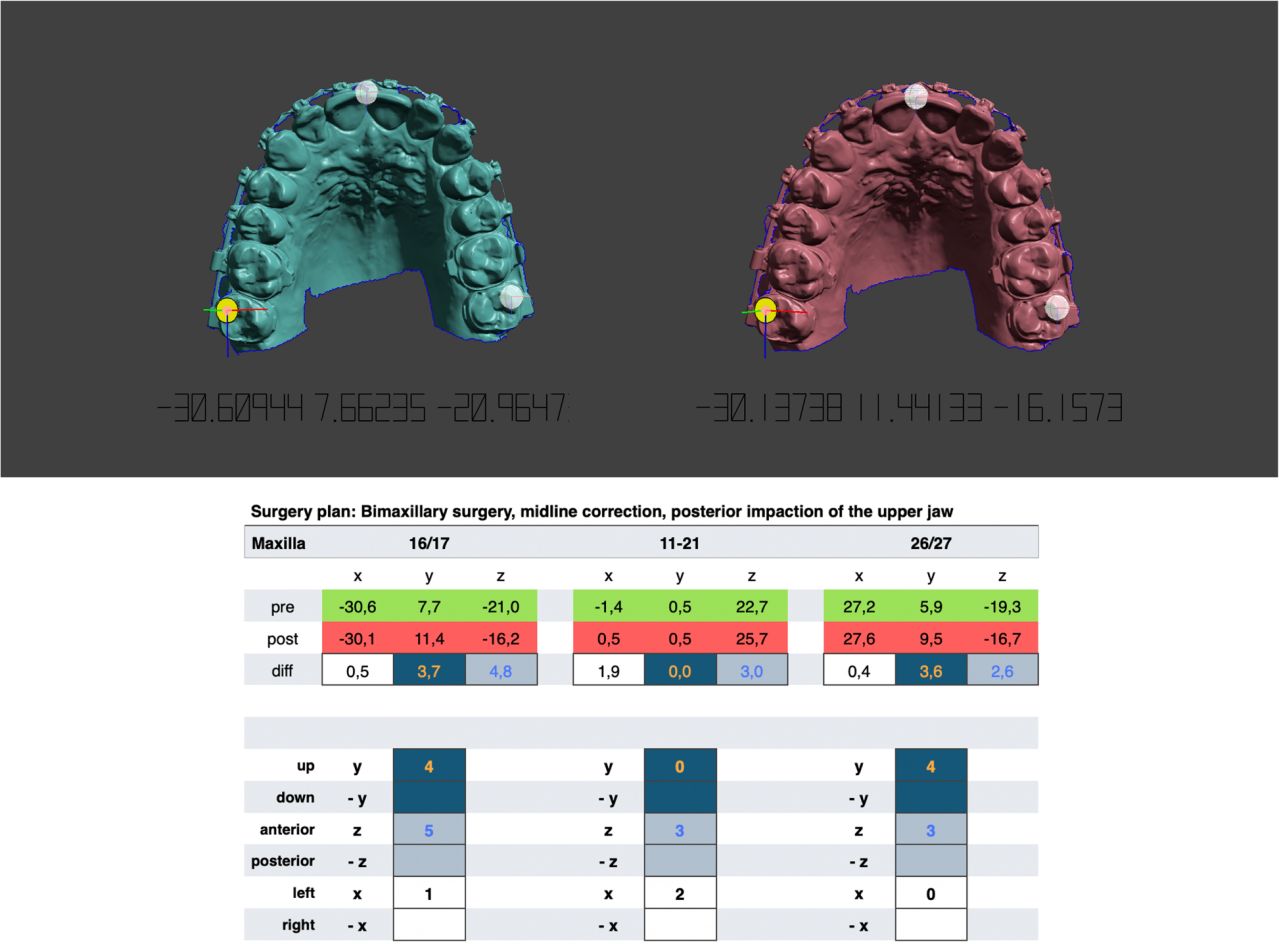
Initial and cloned upper jaw. Measuring point on tooth 17 is selected and the point coordinates measuring tool displays the X/Y/Z coordinates. The differences are calculated by entering the values in a spreadsheet of the planning sheet. In this case the maxilla is asymmetrically advanced by 5 mm on the right side and 3 mm on the left side with a dental midline correction of 2 mm to the left. Simultaneously the jaw is posterior impacted by 4 mm

The lower jaw is moved to its new occlusion in the same way. Unlike plaster, there is no haptic feedback and the meshes can merge into each other. The lack of haptics is compensated visually by highlighting the mesh collisions in colour. So the new occlusal contact points are easy to recognise. After the postoperative positions have been reached, the movements of the individual jaws can be measured. This is done with the point coordinates measuring tool. This measure type allows to query the 3D position of a bounded measuring pivot. Three X/Y/Z coordinates are displayed and are copied into a spreadsheet of the planning protocol. The distances of the pivots between the preoperative and postoperative position are determined and provide information about the displacement of the jaws according to the X/Y/Z axes (Fig. 7). Planning alternatives can be performed by further cloning of the preoperative jaws and the number of plannings are only limited by the storage of the used computer hardware.

Once the optimal movements and positions of the jaws have been determined, the preparation for splint fabrication takes place. Regardless of whether the splints are designed in Meshmixer or a special dental software, an opening rotation must take place in the thickness of the final splint between the upper and lower jaw. For this, the postoperative lower jaw is bound to the hinge axis pivot and the center of transformation is set to the center of the pivot. Depending of the malocclusion an open rotation of approximately two degrees is sufficient.

**Fig. 8:**
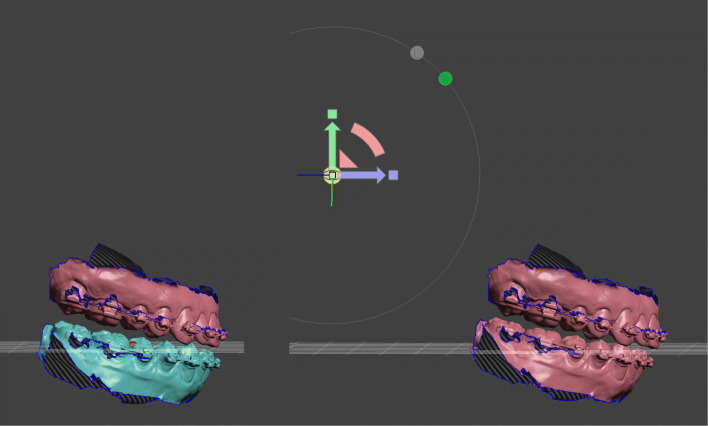
Preparation for splint production for bimaxillary surgery. Intermediate splint: Preoperative lower jaw position with postoperative upper jaw position. Final splint: Postoperative upper jaw position with open rotated postoperative lower jaw position. The amount of open rotation could be adjusted manually by the 3D widget or by the transform tool property panel

**Fig. 9:**
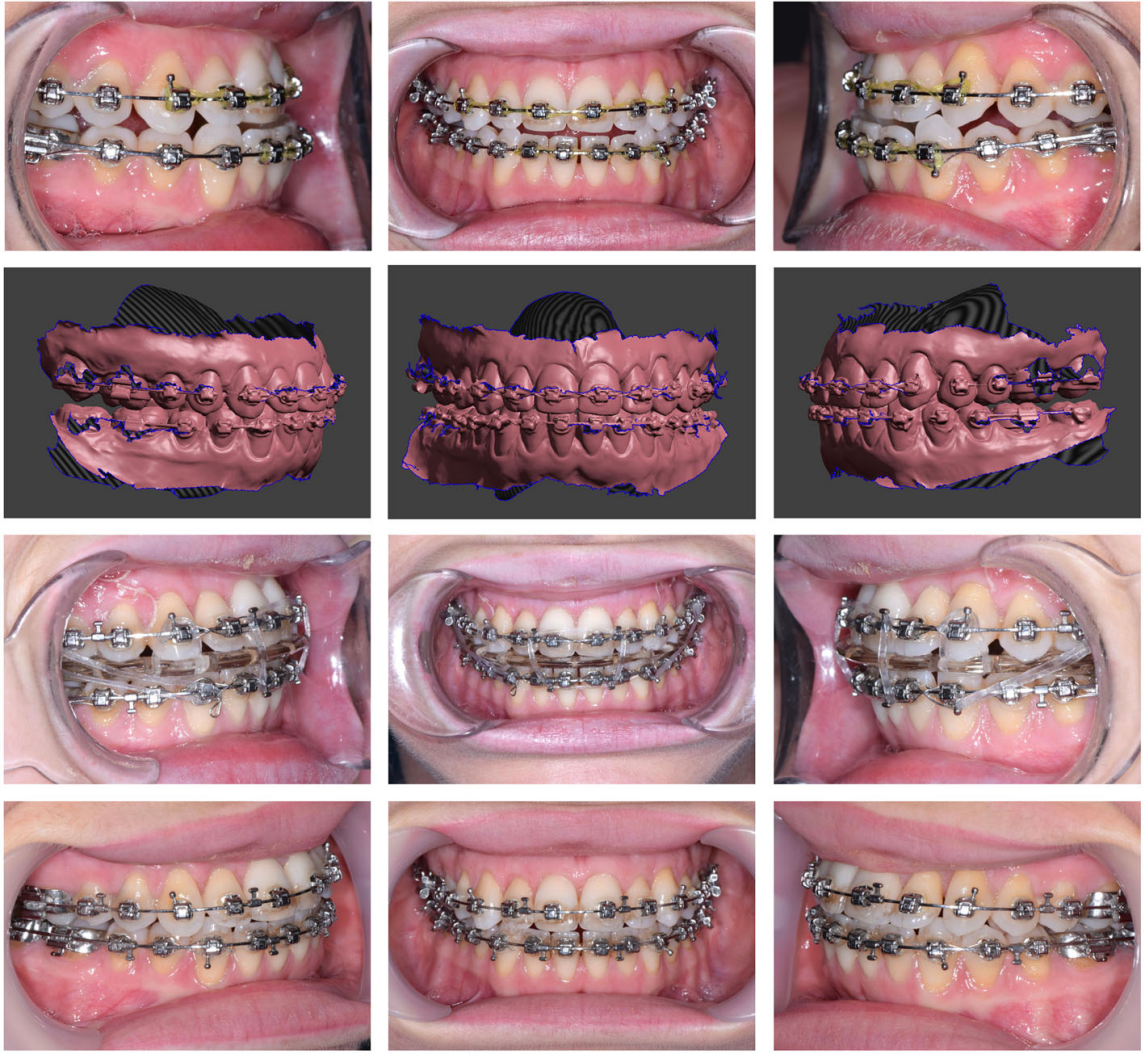
Pre- and postoperative images of a skeletal class III patient. As shown in in Figs. 3–7 the surgery plan was a two-jaw procedure with a maxillary asymmetric advancement and posterior impaction. The lower jaw was a symmetrically set up and back. Upper row: Preoperative occlusion. Second row: Planned occlusion in Meshmixer. Third row: Four weeks postoperative with removable splint and class III elastics. Lower row: Occlusal situation at the appointment for plate removal six month after surgery

If the production of splints outside of Meshmixer is desired, the corresponding STLs must be exported in their relative positions to each other. In case of lower jaw surgery the preoperative maxilla and the postoperative open rotated mandible are exported. Similar applies to single upper jaw surgery. In case of two jaw surgery and for producing the intermediate splint three STL files are exported (Fig. 8): i) the postoperative upper jaw, ii) the unoperated lower jaw, and iii) the postoperative rotated lower jaw. The result of the case used here as an example is shown in Fig. 9.

### Planning protocol

During planning, all necessary information is documented in a special multi-page planning sheet. This sheet contains the cephalometric analysis, the photo analysis, data of the virtual mounting, model surgery movements (Fig. 7), images of pre- and postoperative jaw overlays, an image of the new occlusal contacts, a recommendation how to wear elastics postsurgery, and a 3D representation of the new jaw positions in its final occlusion. This sheet is used by the planning team, the surgeon in the operating theatre and the orthodontist who will carry out the postoperative treatment.

## Results and discussion

The digital planning procedure presented enables all planning scenarios as they are also possible with conventional plaster-based procedures. A significant advantage is that articulator hardware and manual plaster work are no longer required. The full potential can unfold when intraoral scanners and digital splint fabrication are used.

One of the strength of the presented method is the elimination of many error prone laboratory steps. Removing the impression of dental arches attached with brackets and wires can lead to dimensional errors and continues with plaster model fabrication and handling during physical model surgery. Using intraoral scans and direct printing or milling is the most accurate surgical splint production today.

However, the authors’ method is an arbitrary procedure and includes the inaccuracies of conventional orthognathic surgery planning. Arbitrary means, among other things, that the used hinge axis is located 10 mm anterior of the ear rods within a 4-5 mm radius of the true hinge axis [1, 18]. Within this radius the arbritrary hinge axis mounting results in a 10% rate of occlusal errors ≥0.34 mm [18] which is more than acceptable for postoperative orthodontic treatment. Even the purely rotational and therefore non-physiological movement of approx. two degrees for final splint production does not produce any errors that cannot be compensated with postoperative orthodontics.

A further flaw is that the angle between the upper occlusal plane and the AOP is smaller in cephalometric radiographs than in face-bow mounted casts [19], which tends to result in larger upper jaw impactions and more posterior positions of both jaws [1]. Although we worked with metal markers to construct the AOP on the cephalometric radiograph, this error cannot be excluded. Clinical studies quantifying this error are lacking so far. Moreover, focusing planning on the occlusal plane solely runs the risk of not recognizing bony interferences that may result from the displacement of the jaws or jaw segments in both the maxilla and mandible.

The use of a wooden tongue depressor is a standard procedure when assessing occlusal canting [20]. In the clinical application the depressor’s position is influenced by single tooth position and thereby may indicate an incorrect position of the occlusal plane. In addition, when holding the depressor with the teeth, only the anterior part of the dental arch is represented which makes the application in general insufficient. We therefore use cheek retractors to assess the whole upper dental arch on photographs. It is a time feasible and dentition-independent procedure that can be easily used during photographic examination. Not only the dental arch, but also the wire plane, the dental midline and second order crown angulations can be assessed in relation to the interpupillary and midsagittal plane.

Excellent clinical results are achieved with conventional planning and the success of treatment does not depend on planning alone. Proffit et al. [21] were able to show that the stability and predictability of orthognathic surgical procedures depend predominantly on the direction of surgical movement, the type of fixation and the surgical technique used. Two millimetre relaps were defined as the threshold between clinically relevant and non-relevant changes. In an updated paper on the hierarchy of surgical stability Proffit and coworkers noted that a surprisingly large number of patients experience relevant skeletal changes from one to five years post-surgery, when healing is complete [22]. It is very unlikely that these changes are in any way related to the type of planning.

## Conclusion

We believe that arbitrary planning will continue to have its place in the treatment of orthognathic surgery, especially when digital methods can improve the overall process. The method presented can be seen as a cost-effective alternative for patients who do not require technically complex planning.

## Data Availability

Not applicable.

## References

[1] Ellis E, Tharanon W, Gambrell K (1992). Accuracy of face-bow transfer: Effect on surgical prediction and postsurgical result. J Oral Maxillofac Surg.

[2] Imai H, Fujita K, Yamashita Y, Yajima Y, Takasu H, Takeda A, Honda K, Iwai T, Mitsudo K, Ono T, Omura S (2020). Accuracy of mandible-independent maxillary repositioning using pre-bent locking plates: a pilot study,. Int J Oral Maxillofac Surg.

[3] Liczmanski K, Stamm T, Sauerland C, Blanck-Lubarsch M (2020). Accuracy of intraoral scans in the mixed dentition: A prospective non-randomized comparative clinical trial. Head Face Med.

[4] Almutairi T, Naudi K, Nairn N, Ju X, Whitters J, Ayoub A (2018). Replacement of the distorted dentition of the cone-beam computed tomography scans for orthognathic surgery planning. J Oral Maxillofac Surg.

[5] Ehmer U, Austermann KH (1987). Die Rolle des Kieferorthopäden für die Motivation zu chirurgisch-kieferorthopädischen Therapiemaßnahmen. Fortschr Kieferorthop.

[6] Ehmer U, Röhling J, Klang KD, Becker R (1987). A calibrated double cast method for model simulation in surgical orthodontics. Deut Z fur Mund-, Kiefer- und Gesichtschir.

[7] Ehmer U, Rohling J, Dorr K, Becker R (1989). Calibrated double split cast simulations for orthognathic surgery. Int J Adult Orthod Orthognathic Surg.

[8] Ehmer U, Joos U, Flieger S, Wiechmann D (2012). The University Münster model surgery system for Orthognathic surgery. Part I – The idea behind. Head Face Med.

[9] Ehmer U, Joos U, Ziebura T, Flieger S, Wiechmann D (2013). The University Münster model surgery system for orthognathic surgery. Part II – KD-MMS. Head Face Med.

[10] Steinhäuser E, Janson E (1988). Behandlungsaufgaben, Therapieplanung. Kieferorthopädische Chirurgie Band I.

[11] Krenkel C, Lixl G (1991). Model surgical apparatus for planning and simulation of maxillary and mandibular osteotomies. Zahnarztliche Prax.

[12] Erickson K, Bell W, Goldsmith D (1992). Analytical Model Surgery,. Modern Practice in Orthognathic and Reconstructive Surgery.

[13] Schwestka-Polly R, Kubein-Meesenburg D, Luhr HG (1999). Results of the application of the Goettingen concept for three-dimensional repositioning of the maxilla in orthognathic surgery. Mund-, Kiefer- und Gesichtschirurgie : MKG.

[14] Slavicek R (1988). Dr. Rudolf Slavicek on clinical and instrumental functional analysis for diagnosis and treatment planning. Part 1. Interview by Dr. Eugene L. Gottlieb,. J Clin Orthod.

[15] Wood DP, Korne PH (1992). Estimated and true hinge axis: a comparison of condylar displacements. Angle Orthod.

[16] Shildkraut M, Wood DP, Hunter WS (1994). The CR-CO discrepancy and its effect on cephalometric measurements,. Angle Orthod.

[17] Schneider CA, Rasband WS, Eliceiri KW (2012). NIH Image to ImageJ: 25 years of image analysis. Nat Methods.

[18] Morneburg TR, Pröschel PA (2011). Impact of arbitrary and mean transfer of dental casts to the articulator on centric occlusal errors. Clin Oral Inv.

[19] Bailey JO, Nowlin TP (1984). Evaluation of the third point of reference for mounting maxillary casts on the Hanau articulator. J Prosthet Dent.

[20] Kim A, Kim S, Lee H, Oh KS (2020). Serial measurements of facial asymmetry using a wooden tongue depressor in patients with congenital microtia. J Plast Reconstr Aesthet Surg.

[21] Proffit WR, Turvey TA, Phillips C (1996). Orthognathic surgery: a hierarchy of stability,. Int J Adult Orthod Orthognathic Surg.

[22] Proffit WR, Turvey TA, Phillips C (2007). The hierarchy of stability and predictability in orthognathic surgery with rigid fixation: an update and extension,. Head Face Med.

